# Supplementary material for: Motion and teleportation of polar bubbles in low-dimensional ferroelectrics
Source: Nat Commun. 2024 Jan 9;15:412. doi: 10.1038/s41467-023-44639-4 (PMC10776862; doi:10.1038/s41467-023-44639-4)
Supplement: Supplementary file 1 — Supplementary Information [file 41467_2023_44639_MOESM1_ESM.pdf]

Supplementary Information for

"Motion and teleportation of polar bubbles in low-dimensional ferroelectrics"

S. Prokhorenko[1*], Y. Nahas[1], V. Govinden[2], Q. Zhang[2,3**], N. Valanoor[2], L. Bellaiche[1]

[1]Physics Department and Institute for Nanoscience and Engineering, University of Arkansas, Fayetteville, Arkansas 72701, USA

[2]School of Materials Science and Engineering, The University of New South Wales, Sydney, New South Wales 2052, Australia

[3]CSIRO Manufacturing, Lindfield, NSW 2070, Australia

*sprokhor@uark.edu

**peggy.zhang@csiro.au

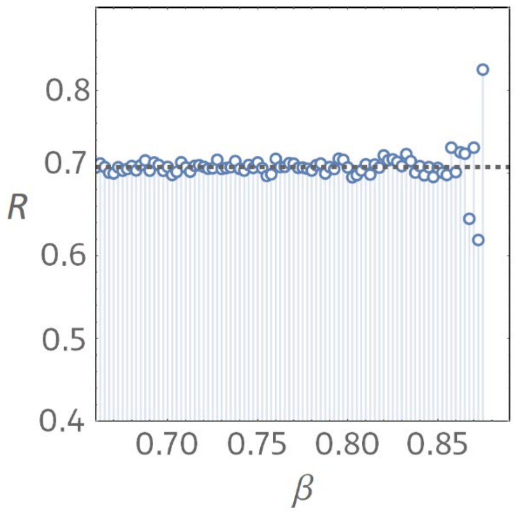

Supplementary Figure 1. **Geometric constraint on the bubble lattice phase.** The dependence of the total switched area at the bub-FE transition on screening obtained from Monte Carlo simulations. The data shows that the bubble lattice state onsets when 69% of the films' area is polarized along the applied bias.

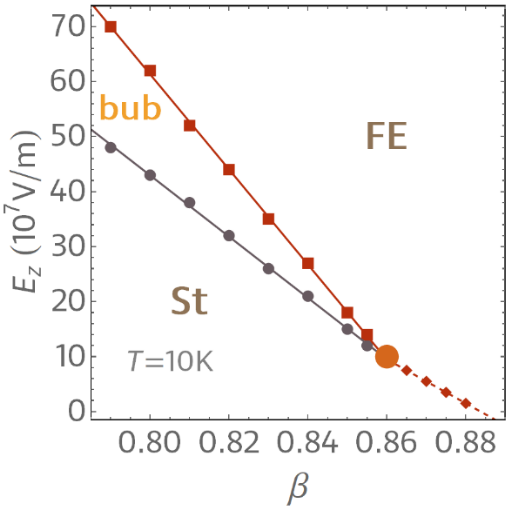

Supplementary Figure 2. **Low temperature phase diagram.** The phase diagram obtained from Monte Carlo simulations at 10K. At this temperature, the transition from the bubble lattice to a homogeneously polarized state is of the 1st order for all considered β values. The orange circle indicates the intersection point of the bub-FE and St-bub lines.

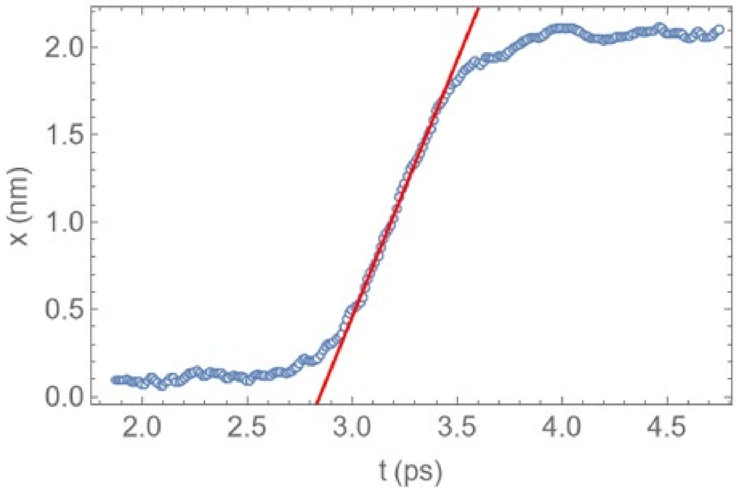

Supplementary Figure 3. Evolution of the bubble position during the continuous motion induced by the PFM tip. The results are obtained using a 64×64×5 supercell at 10K, the bias field of 61×10^7 V/m and the peak driving field magnitude of 30×10^7 V/m. The red line indicates a linear fit used to estimate the bubble velocity.

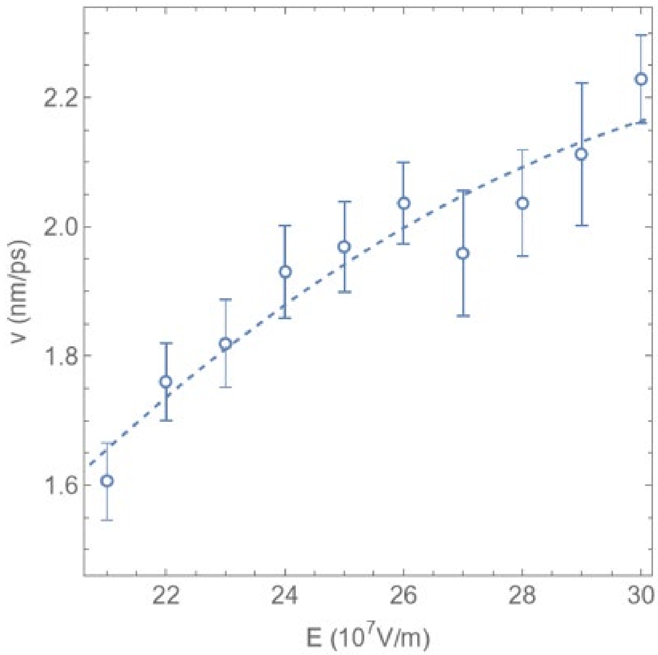

Supplementary Figure 4. Bubble velocity during the continuous motion. The dependence of the bubble velocity v on the driving field magnitude. The results are obtained using a 64x64x5 supercell at 10K and the constant bias field of 61×10^7 V/m. The error bars indicate the standard error computed from a sample of 20 MD simulations for each field value.

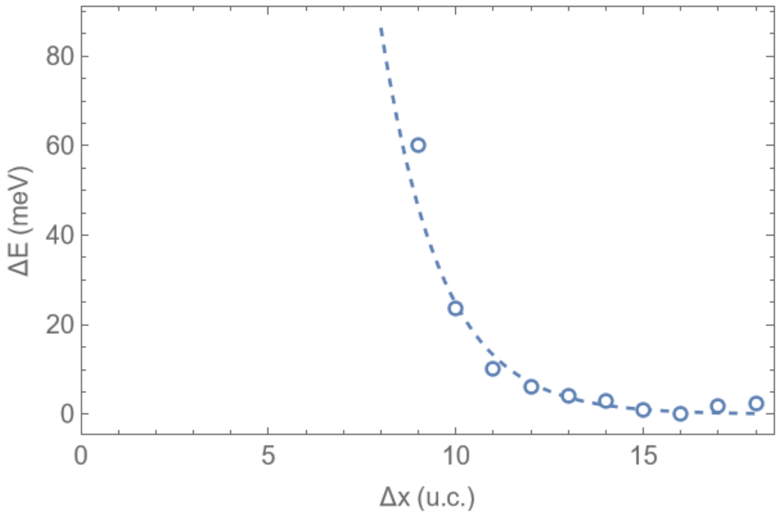

Supplementary Figure 5. Pairwise interaction potential of bubbles. Dependence of the bubble interaction energy ΔE on the bubble separation distance Δx. The results are obtained using a 64×64×5 supercell at 10K and the bias field of 55×10^7 V/m.
