# Supplementary material for: Motion and teleportation of polar bubbles in low-dimensional ferroelectrics
Source: Nat Commun. 2024 Jan 9;15:412. doi: 10.1038/s41467-023-44639-4 (PMC10776862; doi:10.1038/s41467-023-44639-4)
Supplement: Supplementary file 3 — Description of Additional Supplementary Files [file 41467_2023_44639_MOESM3_ESM.pdf]

Description of Additional Supplementary Files

Supplementary Movie 1 Spontaneous bubble dynamics.

Animation of the molecular dynamics evolution of the dipolar structure at 300K, β=0.8 and the bias of 40×10^7 V/m. The total evolution time corresponds to 100 ps with 1 ps between subsequent frames.
