# Supplementary figures and images for: Motion and teleportation of polar bubbles in low-dimensional ferroelectrics
Source: Nat Commun. 2024 Jan 9;15:412. doi: 10.1038/s41467-023-44639-4 (PMC10776862; doi:10.1038/s41467-023-44639-4)

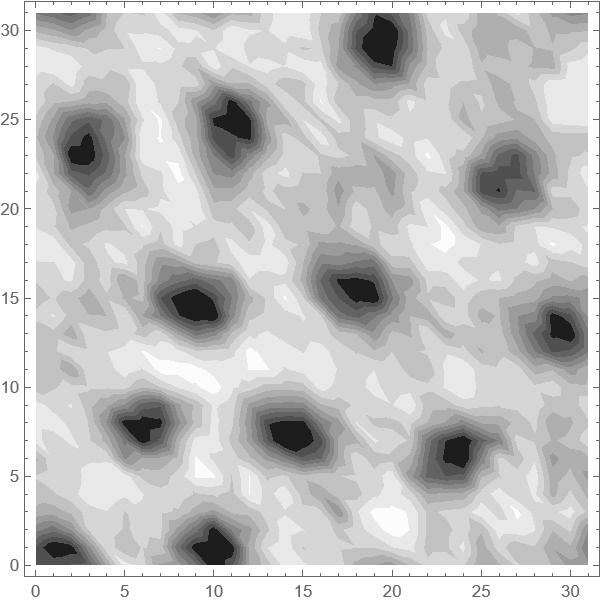

Supplement: Supplementary file 4 — Supplementary Movie 1 [file 41467_2023_44639_MOESM4_ESM.gif]
